# Structural insights into the role of DNA-PK as a master regulator in NHEJ

**DOI:** 10.1007/s42764-021-00047-w

**Published:** 2021-07-23

**Authors:** Siyu Chen, James P. Lees-Miller, Yuan He, Susan P. Lees-Miller

**Affiliations:** 1 Department of Molecular Biosciences, Northwestern University, Evanston, IL USA; 2 Interdisciplinary Biological Sciences Program, Northwestern University, Evanston, IL USA; 3 Department of Biochemistry and Molecular Biology, Cumming School of Medicine, University of Calgary, Calgary, AB Canada; 4 Robson DNA Science Centre, Arnie Charbonneau Cancer Institute, Cumming School of Medicine, University of Calgary, Calgary, AB Canada

**Keywords:** DNA-PKcs, Non-homologous end joining, Cryo-EM, Structural biology, Phosphorylation

## Abstract

DNA-dependent protein kinase catalytic subunit DNA-PKcs/*PRKDC* is the largest serine/threonine protein kinase of the phosphatidyl inositol 3-kinase-like protein kinase (PIKK) family and is the most highly expressed PIKK in human cells. With its DNA-binding partner Ku70/80, DNA-PKcs is required for regulated and efficient repair of ionizing radiation-induced DNA double-strand breaks via the non-homologous end joining (NHEJ) pathway. Loss of DNA-PKcs or other NHEJ factors leads to radiation sensitivity and unrepaired DNA double-strand breaks (DSBs), as well as defects in V(D)J recombination and immune defects. In this review, we highlight the contributions of the late Dr. Carl W. Anderson to the discovery and early characterization of DNA-PK. We furthermore build upon his foundational work to provide recent insights into the structure of NHEJ synaptic complexes, an evolutionarily conserved and functionally important YRPD motif, and the role of DNA-PKcs and its phosphorylation in NHEJ. The combined results identify DNA-PKcs as a master regulator that is activated by its detection of two double-strand DNA ends for a cascade of phosphorylation events that provide specificity and efficiency in assembling the synaptic complex for NHEJ.

## Introduction

The phosphatidyl inositol 3-kinase-like protein kinases (PIKKs) are a family of serine/threonine protein kinases with similarity in their kinase domains to the lipid kinase *PIK3CA*, the catalytic subunit of phosphatidyl inositol 3-kinase, PI3K. However, the PIKKs are serine/threonine protein kinases and three of them, DNA-dependent protein kinase catalytic subunit (DNA-PKcs, gene name *PRKDC*), Ataxia telangiectasia mutated (ATM) and ATM and Rad3 related (ATR), play important roles in the cellular response to DNA damage (Blackford & Jackson, 2017). In this review, we note and pay tribute to the scientist who first discovered the DNA-activated protein kinase, Dr. Carl W. Anderson, who sadly passed away October 20, 2020, aged 76. We will then place Anderson’s discoveries into the context of breakthrough advances in understanding the structure and function of the largest member of the PIKK family, DNA-PKcs.

Carl spent a large part of his career at the Biology Department at Brookhaven National Laboratory, Upton, NY, where he was Chair from 1999 to his retirement in 2011. In 1985, Carl carried out a sabbatical in the lab of Dr. Tim Hunt at Cambridge University, UK where he planned to work on the RNA-dependent protein kinase. However, the RNA they purchased for their experiments was contaminated with double-stranded (ds)DNA, leading to the serendipitous discovery that human cells contain a DNA-activated protein kinase that phosphorylates the 90-kDa heat shock protein (Hsp90) and other proteins in HeLa cell extracts (Walker et al., 1985). One of the authors of this review, S. P. Lees-Miller, was fortunate to join Carl’s lab as a post-doctoral fellow to attempt to purify and characterize this putative DNA-activated protein kinase. In his lab, we showed that the DNA-activated protein kinase phosphorylates the α isoform of Hsp90 on two N-terminal threonine residues that are each followed by a glutamine, in the sequence PEETQTQDQPME (Lees-Miller & Anderson, 1989). Thus, Hsp90-α was the first PIKK substrate shown to be phosphorylated on an SQ/TQ motif, now recognized as a characteristic signature of many PIKK substrates (O'Neill et al., 2000; Schlam-Babayov et al., 2021).

Using Hsp90 as our substrate, we attempted to purify the DNA-activated protein kinase and showed that its activity was associated with a large polypeptide we called p350 (now known as DNA-PKcs) and the dsDNA binding Ku70/80 heterodimer (Lees-Miller et al., 1990). p350 was independently identified as a DNA-activated protein kinase by Dr. Tim Carter of St. John’s University, Jamaica, NY (Carter et al., 1990), and the groups of Drs. Steve Jackson (Gottlieb & Jackson, 1993; Jackson et al., 1990) and Bill Dynan (Dvir et al., 1992, 1993). As part of these efforts, Carl made important contributions to the cloning of the DNA-PKcs cDNA (Hartley et al., 1995), as well as its genomic location on 8q11 (Sipley et al., 1995) and reported that DNA-PKcs shares a bi-directional promoter with replication factor MCM2 (Connelly et al., 1998). Carl also had a long-standing interest in p53 (Nguyen et al., 2014, 2018) and showed that DNA-PK phosphorylates p53 on serine 15 in vitro, another SQ site, establishing the first quantitative assay for DNA-PK kinase activity (Lees-Miller et al., 1992).

In sum, Carl made many important contributions to the discovery and function of DNA-PK and the regulation of p53. He was a kind and generous man. He was a great mentor. He will be sorely missed by his many friends and colleagues. Fortunately, his many contributions to science and in particular to our understanding of DNA-PKcs will live on. In the subsequent sections, we build upon Carl’s foundational results to provide the integrated structural and functional characterization of DNA-PK and its role in NHEJ.

## Repair and non-repair functions of DNA-PKcs

**Fig. 1:**
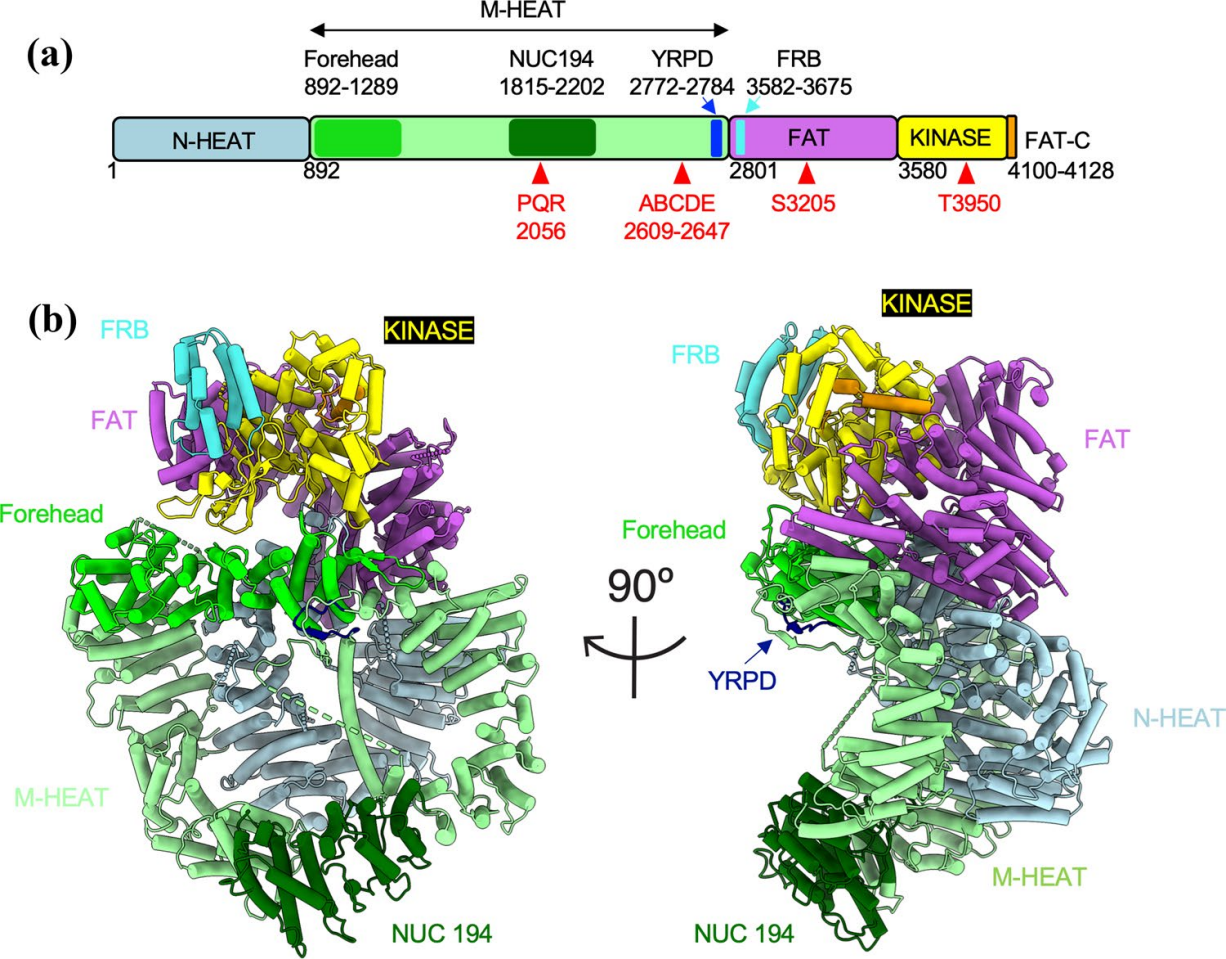
Structure of DNA-PKcs. **A** Schematic of DNA-PKcs showing the N-HEAT domain (residues 1–891, light blue), the M-HEAT domain (residues 892–2800, light green), the FAT domain (residues 2801–3579, purple), the kinase domain (3580–4099, yellow) and the FATC domain (4100–4128, orange). Also shown is the position of the conserved forehead domain (892–1289, bright green) and the NUC194 domain (residues 1815–2202, dark green), the YRPD motif (residues 2772–2784, dark blue), the FRB domain (residues 3582–3675, bright blue) and the positions of the PQR, ABCDE, S3205 and T3950 phosphorylation sites (red triangles). **B** Two views of DNA-PKcs (from PDB 7LT3) (Chen et al., 2021a), rotated by 90°, colored as in **A**. The YRPD motif is shown in dark blue, most clearly visible in the side view, indicated by an arrow. See Lees-Miller et al., (2020) for conservation of amino acids in the forehead and NUC194 domains in DNA-PKcs from eukaryotes

**Fig. 2:**
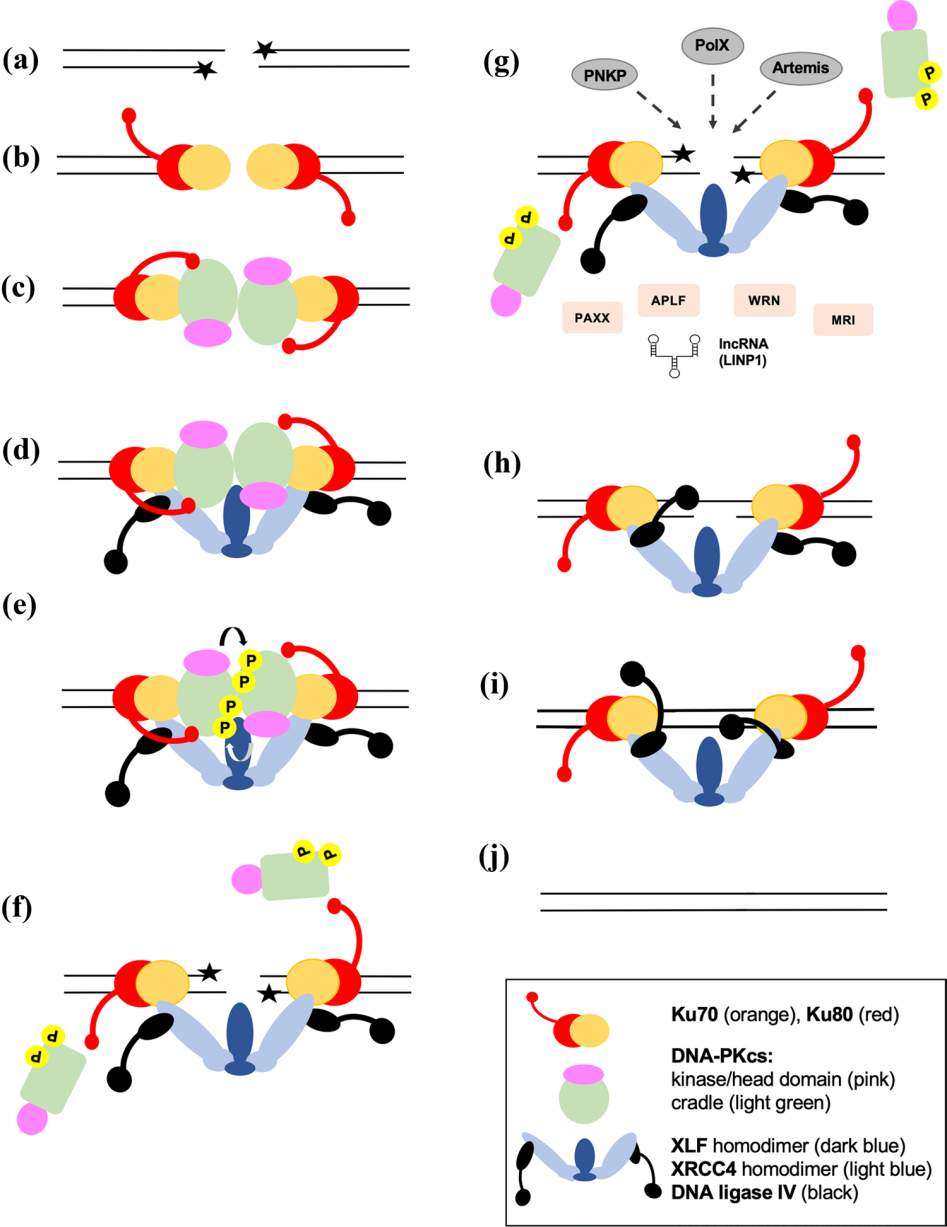
Model for NHEJ. **A** IR induces DSBs, often with damaged ends that contain non-ligatable end groups (stars). **B **A Ku heterodimer (composed of Ku70, orange; Ku80, red) binds to each end of the DSB and **C** recruits DNA-PKcs (shown with head /kinase-FAT-FATC domain in pink and circular cradle in green) to form a synaptic complex. **D** The Ku complex also recruits a single homodimer of XLF (dark blue) that interacts via its head domain with two XRCC4 homodimers (light blue). The coiled-coil domain of XRCC4 interacts with tandem BRCT domains in the C-terminal region of DNA ligase IV (black). This forms a long-range synaptic complex as reported in Chen et al. (2021a). **E** Autophosphorylation of DNA-PKcs at ABCDE and possibly other sites, likely in trans leads to a conformational change that causes release of the DSB ends by DNA-PKcs. **F** autophosphorylated DNA-PKcs dissociates from the complex providing access to processing enzymes such as PNKP, Artemis and DNA polymerases mu and lambda which remove non-ligatable end groups and fill in gaps (**G**). **H** and **I** each DNA ligase IV is tethered to an XRCC4 homodimer through its C-terminal BRCT domains while the N-terminal catalytic domain is attached via a flexible linker region. This allows the catalytic domains to access the DSB ends while Ligase IV remains tethered to the synaptic complex. Each DNA Ligase IV repairs a single-strand break so that the two breaks are repaired sequentially. **J** The break is repaired restoring genome integrity. **G** shows additional NHEJ factors such as PAXX, APLF, WRN, Cyren/MRI and long non-coding RNA which may also be involved. The precise order of recruitment and dissociation of the NHEJ proteins is unknown

**Fig. 3:**
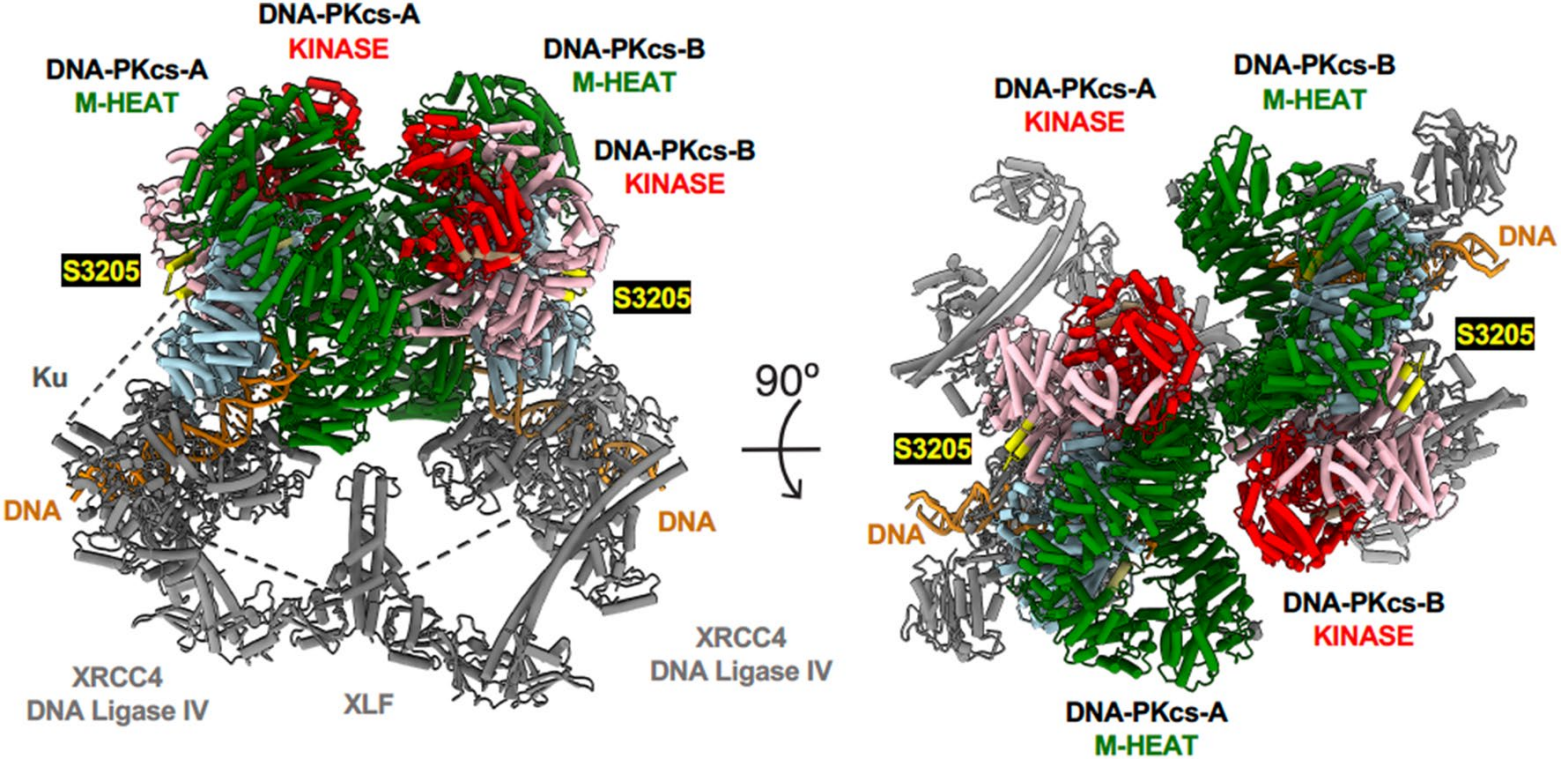
Model for long-range synaptic complex. The long-range synaptic complex (PDB: 7LT3) (Chen et al., 2021a) was generated using ChimeraX 1.1.1 (Pettersen et al., 2021). The N-HEAT domain is shown in light blue, the M-HEAT in green and the kinase domain in red. The FAT domain is in pink and the FATC in tan. dsDNA is shown in dark orange. The Ku heterodimer, XLF and XRCC4-Lig4 are shown in grey. The structure on the right is rotated by 90° to reveal the relative positions of the kinase domains (red) and circular cradles (green) in the opposing DNA-PKcs molecules. The location of the ATM-dependent phosphorylation site S3205 is shown in yellow

**Fig. 4:**
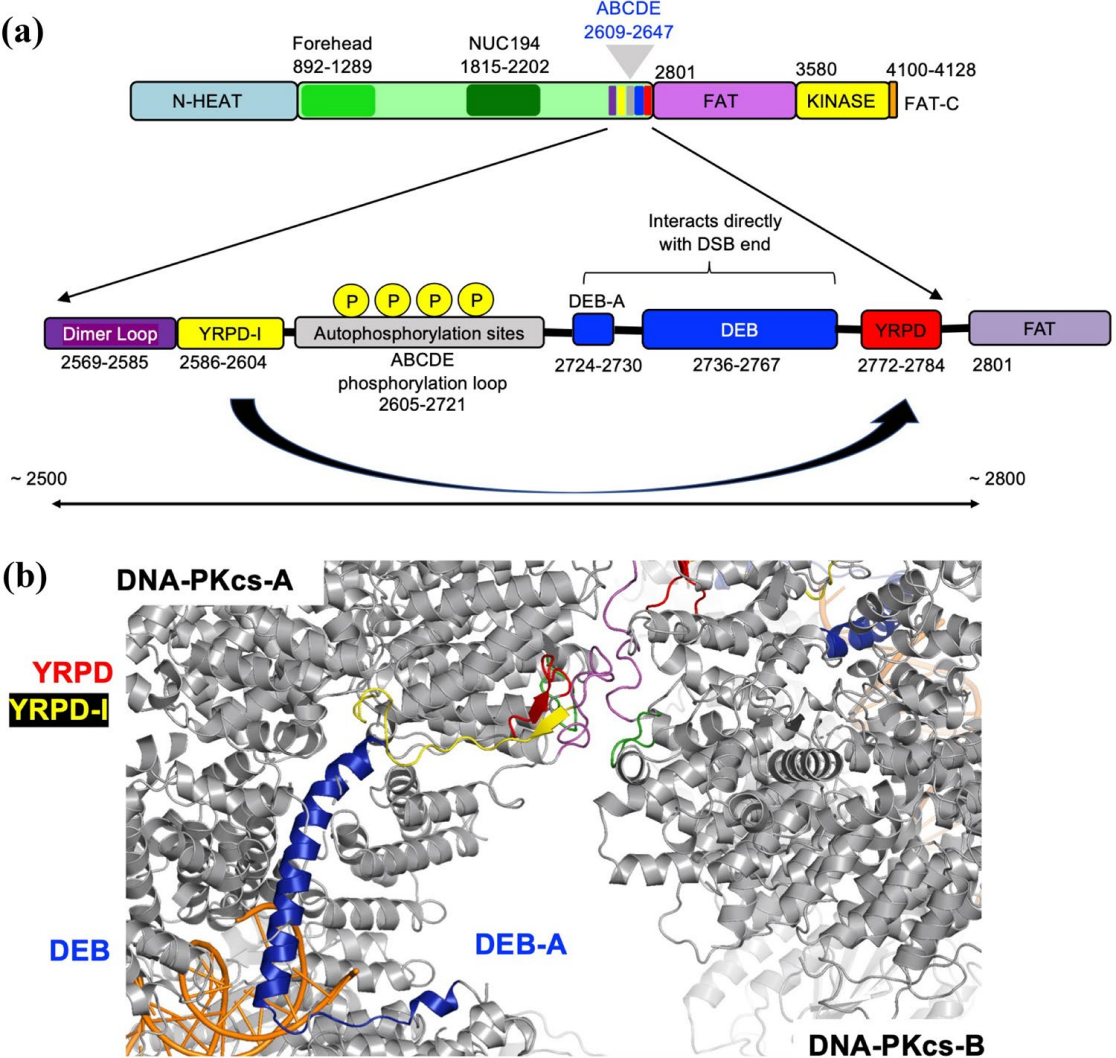
Cartoon showing regulatory sequences located in DNA-PKcs between 2500 and 2800. **A** Schematic showing the region of DNA-PKcs between amino acids 2500 and 2800 that regulates the synaptic interface. The dimerization loop (residues 2569–2585), the YRPD-interacting motif (2586–2604), the DNA End Binding (DEB) helix (2736–2767) with DEB-appended (DEB-A, residues 2724–2730) and a short linker (2731–2735), and the YRPD motif (2722–2784) are from Chen et al. (2021a). DEB-A, the linker and 37 amino acid DEB form a basic surface that interacts directly with the ends of the DSB (Chen et al., 2021a). **B** Close up view of interaction interface between two DNA-PKcs molecules, labeled DNA-PKcs-A and DNA-PKcs-B, showing the forehead loop (residues 896–903, green), the dimerization loop (2569–2585, purple), the YRPD-I (residues 2586–2604, yellow), the YRPD (2774–2784, red) and the DEB-A, linker DEB (2724–2767, blue). The position of the DEB is maintained through interaction of the YRPD and the YRPD-I, shown as yellow and red beta sheets respectively, the dimerization loop and the forehead loop. The disordered loop containing the ABCDE autophosphorylation sites (residues 2605–2721) is absent from the structures and is not shown

DNA-PKcs is a 4128 amino acid polypeptide composed of a large N-terminal HEAT (Huntingtin, Elongation Factor 3, protein phosphatase 2 A subunit, TOR) repeat containing domain, a region of conservation between FRAP, ATM and TRRAP (the FAT domain) followed by the kinase domain and a short C-terminal region termed the FATC domain (Fig. 1). The N-terminal region is subdivided into an N-terminal HEAT repeat domain (N-HEAT), which is unique to DNA-PKcs, and the middle (M-HEAT) domain which forms an α-solenoid, also called the circular cradle (Baretic et al., 2019; Sibanda et al., 2017) (Fig. 1). DNA-PKcs interacts with Ku70/80 in the presence of dsDNA to form the active DNA-dependent protein kinase holoenzyme DNA-PK, and is required for non-homologous end joining (NHEJ), the major pathway for the repair of ionizing radiation (IR)-induced DNA double-strand breaks (DSBs) in human cells (Yue et al., 2020; Zhao et al., 2020a). In NHEJ, the Ku heterodimer binds directly to DSB ends (Fig. 2a, b), then translocates inwards, allowing DNA-PKcs to interact with the DSB end (Yoo & Dynan, 1999) (Fig. 2a, c). This complex, composed of two distinct DNA-PK complexes assembled on the two adjacent DSB ends, is often termed a synaptic complex (Fig. 2c) and serves to tether and protect the DSB ends from inappropriate degradation or rejoining. IR-induced DSBs frequently contain non-ligatable end groups (indicated by stars in Fig. 2a) and other forms of DNA damage that must be removed or repaired prior to ligation (Menon & Povirk, 2016). This is thought to be carried out by processing enzymes such as the nuclease Artemis, the 3ʹ-DNA phosphatase/5ʹ-DNA kinase polynucleotide kinase phosphatase (PNKP) and other factors (Ghosh & Raghavan, 2021) (Fig. 2g). Ligatable ends are then rejoined by the XLF-XRCC4-DNA ligase IV complex (Menon & Povirk, 2016; Wang & Lees-Miller, 2013; Zhao et al., 2020a) (Fig. 2h–j). Thus, NHEJ can be considered to proceed in four distinct phases. (1) Detection of the DSB ends by the Ku70/80 heterodimer; (2) recruitment of DNA-PKcs and other proteins to tether the DSB ends together and protect them from nucleolytic digestion (Fig. 2c–e); (3) removal of non-ligatable end groups (Fig. 2g) and (4) ligation of the DSB ends (Fig. 2h–j). However, the precise order of recruitment of NHEJ factors to the DSB is still unclear. Indeed, rather than taking place sequentially, it has been proposed that the NHEJ proteins form a multi-protein assembly at the DSB (Wang & Lees-Miller, 2013; Williams et al., 2014; Yano & Chen, 2008). Assembly of such a complex could have advantages over a sequential pathway in that the DSB ends would be held within the protein complex, protected from inappropriate end joining or nuclease activity, that hand over from one component to another could occur more efficiently and that the complex might maintain some function even if an individual component was missing or mutated, as has been proposed in other DNA repair systems (Hitomi et al., 2007).

Within this NHEJ complex, DNA-PKcs is emerging as a critical master regulator. Interaction of DNA-PKcs with Ku activates its kinase activity and helps tether DSB ends in the initial synaptic complex. As discussed in detail below, DNA-PKcs autophosphorylation results in dissociation of DNA-PKcs from the DSB ends allowing processing enzymes such as Artemis and PNKP (Fig. 2e–g) and ultimately DNA ligase IV to access the DSB ends and complete repair (Fig. 2h–j). DNA-PKcs and Artemis are also required for opening DNA hairpin coding ends during V(D)J recombination and cells lacking DNA-PKcs, Ku, XRCC4 or DNA ligase IV are radiation sensitive and defective in NHEJ and V(D)J recombination (Pannunzio et al., 2018). Recently, additional roles for DNA-PKcs, outside DSB repair have been reported (Yue et al., 2020), including mitosis (Douglas et al., 2014, 2020; Jette & Lees-Miller, 2015; Lee et al., 2011), transcription (Goodwin et al., 2015; Ju et al., 2006), RNA processing (Shao et al., 2020), metastasis (Goodwin et al., 2015), metabolism (Chung, 2018; Park et al., 2017), the innate immune response (Yang et al., 2020) and HIV replication (Anisenko et al., 2020), but its most well-characterized role is in NHEJ.

## DNA-PKcs autophosphorylation in NHEJ

DNA-PKcs phosphorylates many NHEJ proteins in vitro including Ku70 and Ku80 (Chan et al., 1999; Douglas et al., 2005), XRCC4 (Yu et al., 2003), XLF (Yu et al., 2008), DNA Ligase IV (Wang et al., 2004), PNKP (Zolner et al., 2011), APLF (Hammel et al., 2016) and Artemis (Goodarzi et al., 2006) but the functional impact of these phosphorylation events is, in many cases, unclear as ablation of DNA-PKcs phosphorylation sites had little effect on cell survival after IR or on V(D)J recombination (Douglas et al., 2005; Yu et al., 2003, 2008). Moreover, phosphorylation of PNKP (Segal-Raz et al., 2011; Zolner et al., 2011), Artemis (Chen et al., 2005b) and APLF (Macrae et al., 2008) at the same sites is largely ATM-dependent in cells, indicating extensive crosstalk between the DNA-damage-induced PIKKs (Schlam-Babayov et al., 2021).

One of the most well-characterized in vitro and in vivo substrates of DNA-PKcs is itself. DNA-PK undergoes autophosphorylation in vitro and autophosphorylation correlates with loss of DNA-PK kinase activity and dissociation of DNA-PKcs from Ku (Chan & Lees-Miller, 1996). DNA-PKcs autophosphorylation sites include T2609, S2612, T2620, S2624, T2638 and T2647 in the M-HEAT domain (together called the ABCDE cluster), S3205 in the FAT domain, and T3950 in the kinase domain (Chan et al., 2002; Douglas et al., 2002, 2007) (Fig. 1A). Serine 2056 in the HEAT repeat region, part of the PQR cluster, was also identified as an in vitro and in vivo phosphorylation site (Chen et al., 2005a; Cui et al., 2005) and both ABCDE and PQR phosphorylation clusters are important for NHEJ and V(D)J recombination (Convery et al., 2005; Cui et al., 2005; Ding et al., 2003; Jiang et al., 2015; Neal et al., 2011). Biochemical assays using biotinylated DNA pull-down assays with purified proteins confirmed that phosphorylation disrupts the interaction of wt-DNA-PKcs with DNA-bound Ku, while DNA-PKcs in which the ABCDE sites have been mutated to alanine was less effective at phosphorylation-induced dissociation (Hammel et al., 2010; Jette & Lees-Miller, 2015). Together, these studies suggested that DNA-PKcs is recruited to DSBs through its interaction with Ku and that it undergoes autophosphorylation and subsequent release, thus allowing other NHEJ proteins to access the DSB ends to facilitate processing and ligation.

The first suggestion that ABCDE phosphorylation facilitates release of DNA-PKcs from Ku bound DNA in vivo was the observation that DNA-PKcs with all six sites blocked by alanine substitution imparts a more dramatic radiosensitization than cells completely lacking DNA-PKcs (Ding et al., 2003). This was explained by the observation that DNA-PKcs with ABCDE sites blocked remains associated with chromatin for much longer after IR than wild-type DNA-PKcs (Cui et al., 2005). Further support for this model came from laser micro-irradiation experiments showing that whereas wild-type DNA-PKcs was released from sites of DNA damage with a half-life of about 1 h, over 60% of DNA-PKcs in which the S2056, T2609, S2612, T2620, S2624, T2638 and T2647 were mutated to alanine as well as kinase-dead DNA-PKcs were retained at DSB sites for at least 2 h (Uematsu et al., 2007). Given the accumulating evidence of DNA-PKcs’ importance in NHEJ, we re-examined DNA-PKcs evolution. We discovered it is more widespread and ancient than previously supposed and uncovered a high conserved motif that informs cryo-EM structures (Lees-Miller et al., 2020).

## Evolutionary conservation of DNA-PKcs

An impediment to studying the cellular function of DNA-PKcs is its size, 4128 amino acids, making it difficult to express by recombinant means. In addition, unlike ATM and ATR, DNA-PKcs is absent from many model organisms including *Drosophila melanogaster*, *Caenorhabditis elegans*, *Saccharomyces cerevisiae*, *Schizosaccharomyces pombe* and *Arabidopsis thaliana*. This has led to the assumption that DNA-PKcs is a vertebrate-specific PIKK. However, the literature contains reports of putative DNA-PKcs homologues in Arthropods (Dore et al., 2004), *Dictyostelium discoideum* (Block & Lees-Miller, 2005; Pears & Lakin, 2014) and a variety of invertebrates and plants (Elias-Villalobos et al., 2019). To investigate these claims in more detail, we searched NCBI databases for putative DNA-PKcs/*PRKDC* homologues and used multiple sequence analysis to identify conserved amino acids (Lees-Miller et al., 2020). We showed that DNA-PKcs is present in a wide diversity of eukaryotes including Arthropods, Echinoderms, Molluscs, Annelids, Nematodes, Cnidaria, oomycetes, plants, fungi, protozoa and amoeba (Lees-Miller et al., 2020). Yet, DNA-PKcs was not present uniformly within all phyla. For example, although it was present in fungi of phyla Mucoromycota and Chytridiomycota, it was absent from the Dikarya, a subkingdom that includes the yeasts *S. cerevisiae*, *S. pombe* and mushrooms. Similarly, although DNA-PKcs is present in Enoplean nematodes such as Trichinella and Trichuris, it is absent in Chromadorea, which includes *C. elegans*. Furthermore, although DNA-PKcs is present in many plants including green algae, liverworts, mosses, ferns, cycads, and conifers such as pine, fir and spruce, it is absent from flowering plants, including *Arabidopsis thaliana*. Within the insect order Diptera, DNA-PKcs is present in the suborder Nematocera including mosquitoes and midges, but absent in fruit flies from the suborder Brachycera (such as *Drosophila melanogaster*)*.* Why so many model organisms lack DNA-PKcs is unclear, however, these data reveal that DNA-PKcs/*PRKDC* has a far more widespread and ancient lineage than previously supposed (Lees-Miller et al., 2020).

Analysis of DNA-PKcs sequence across species and phyla allowed us to identify regions of high amino acid conservation that likely maintained important functional roles. These included a sequence in the forehead domain at the beginning of the M-HEAT domain (residues 893–1289) and a region between residues 2772 and 2784 in the M-HEAT domain, immediately following the disordered loop containing the ABCDE phosphorylation sites and immediately prior to the FAT domain. Remarkably, in this region, which we termed the YRPD motif, the residues YRxGxxPD were invariant in DNA-PKcs through vertebrates, invertebrates, oomycetes (mildew), amoeba and plants (Lees-Miller et al., 2020), suggesting that they are critical for DNA-PKcs function.

## Structures of NHEJ synaptic complexes

Over the past decade, elegant structural studies have revealed the structure of DNA-PKcs alone and in complex with Ku70/80 and dsDNA (Chaplin et al., 2021; Chen et al., 2021b; Sharif et al., 2017; Sibanda et al., 2010, 2017; Yin et al., 2017). The FAT-kinase and FATC domains form a head or crown at the top of the molecule (represented in pink in Fig. 2), while the N and M-HEAT domains form 2 α-solenoid rings, often referred to as the circular cradle (shown in green in Fig. 2). Double-stranded-DNA, bound within the pre-formed ring of Ku70/80 enters from the back and the base of DNA-PKcs, between the N and M-HEAT repeat rings such that the DSB end is positioned within its central cavity (Chen et al., 2021b; Yin et al., 2017). While these structures were extremely informative, they were unable to provide information on the location of the flexible loop containing the ABCDE phosphorylation sites. In addition, none of the available structures of DNA-PKcs alone or DNA-PK holoenzyme addressed the structure of the proposed NHEJ synaptic complexes, where one molecule of Ku and one molecule of DNA-PKcs is positioned on each side of the DSB (Fig. 2c).

Clues to the structure of NHEJ synaptic complexes came from single molecule (sm) FRET studies in *Xenopus* egg extracts which showed that synapsis occurs in two stages. The first resulted in complex in which the two DSB ends face each other but are offset by more than 100 Å and a second in which the DSBs were more closely aligned (Graham et al., 2016). These were referred to as long-range and short-range complexes, respectively (Graham et al., 2016). Further support for the existence of a long-range complex came from structural mass spectrometry using hydrogen–deuterium exchange and cross-linking where two DNA-PK holoenzymes were shown to form a synaptic complex with the DSB ends staggered, approximately 100 Å apart (Hepburn et al., 2020). Hydrogen–deuterium exchange and cross-linking mass spectrometry studies also indicated that the path of dsDNA within DNA-PKcs was blocked by a flexible “plug domain”, corresponding to residues 2577–2773, i.e., encompassing the ABCDE phosphorylation cluster (Hepburn et al., 2020), however, at 13 Å, precisely how this plug domain functioned was unclear.

To better understand the nature of NHEJ synaptic complexes and the mechanism of NHEJ, we assembled Ku70/80 and DNA-PKcs on dsDNA, then added XLF and X4-L4 and determined the structures of synaptic complexes using cryo-EM (Chen et al., 2021a). We characterized a complex composed of DNA-PKcs, Ku70/80 and dsDNA held by a scaffold containing two XRCC4 homodimers, each bound to one molecule of Lig4 and linked via their head domains by a single XLF homodimer (Fig. 3). Since the DSB ends in this complex were staggered and ~ 115 Å apart (Chen et al., 2021a), we referred to this as the long-range synaptic complex due to its similarities with complexes inferred from FRET (Graham et al., 2016) and mass spectrometry (Hepburn et al., 2020). The stoichiometry of the complex was also consistent with quantitative smFRET data, indicating a single XLF homodimer linked to two XRCC4-Ligase IV molecules (Graham et al., 2018). The DSB ends were located within the central cavity of each DNA-PKcs, moreover, their path was blocked by a DNA end binding (DEB) helix motif composed of DEB-appended helix (DEB-A, residues 2724–2730), a 6 amino acid linker and a 37 amino acid DEB helix (residues 2736–2767) that directly interact with the DSB ends. Moreover, the highly conserved YRPD motif (residues 2772–2784) was shown to form a beta-sheet that interacts with an anti-parallel beta-sheet formed by the YRPD-interacting region (YRPD-I, residues 2586–2604) (Fig. 4).

From the structures, we suggest that two DNA-PKcs molecules interact within a long-range synaptic complex such that autophosphorylation of the ABCDE and PQR phosphorylation sites occurs in trans (Meek et al., 2007), leading to the release of the DSB ends from the DEB/DEB-A motif by charge repulsion, and dissociation of DNA-PKcs. Basic amino acids in the DEB-A and DEB helices are highly conserved (Lees-Miller et al., 2020), indicating the importance of this DNA end binding mechanism. In line with these observations, DNA-PKcs in the long-range complex is captured in its activated state. Thus, we propose that autophosphorylation results in dissociation of DNA-PKcs from the DSB ends, while the Ku-XLF-XRCC4-Ligase IV scaffold retains and aligns the DNA ends for subsequent processing and ligation. However, we cannot exclude the possibility that autophosphorylation can also occur in cis, and that other phosphorylation events are involved. Equally, the coordination of autophosphorylation, end-processing and ligation needs to be highly regulated, and the precise mechanism is yet to be resolved.

**Fig. 5:**
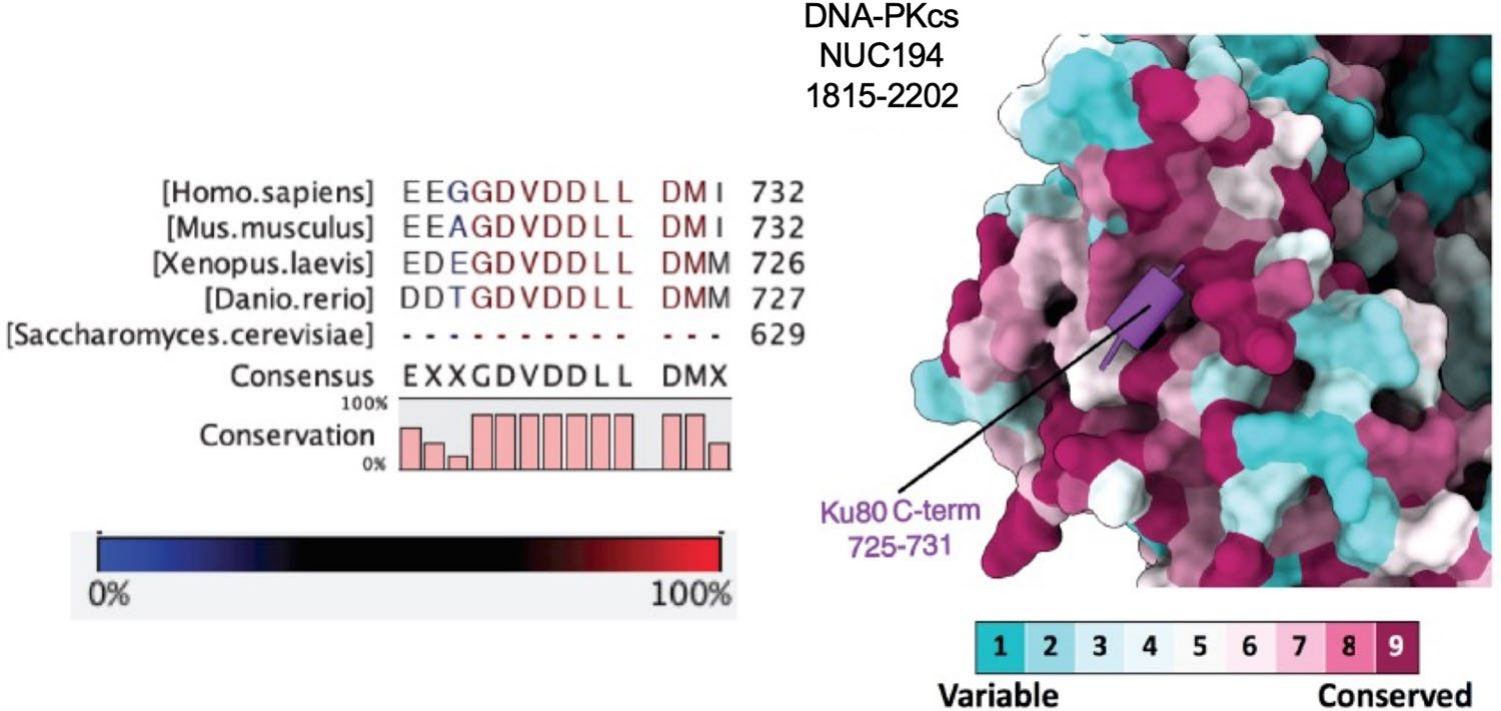
Interaction of the C-terminal region of Ku80 (residues 725–731) with the NUC194 domain of DNA-PKcs. The amino acid sequence VDDLLDM at the extreme C-terminus of Ku80 interacts directly with DNA-PKcs (Falck et al., 2005). In the long-range complex (Chen et al., 2021a), this sequence forms an alpha-helix (purple) that interacts with the NUC194 domain (residues 1815–2202) of DNA-PKcs. Conserved residues within the NUC194 domain of vertebrate model organisms are shown in magenta and variable amino acids are shown in teal

**Fig. 6:**
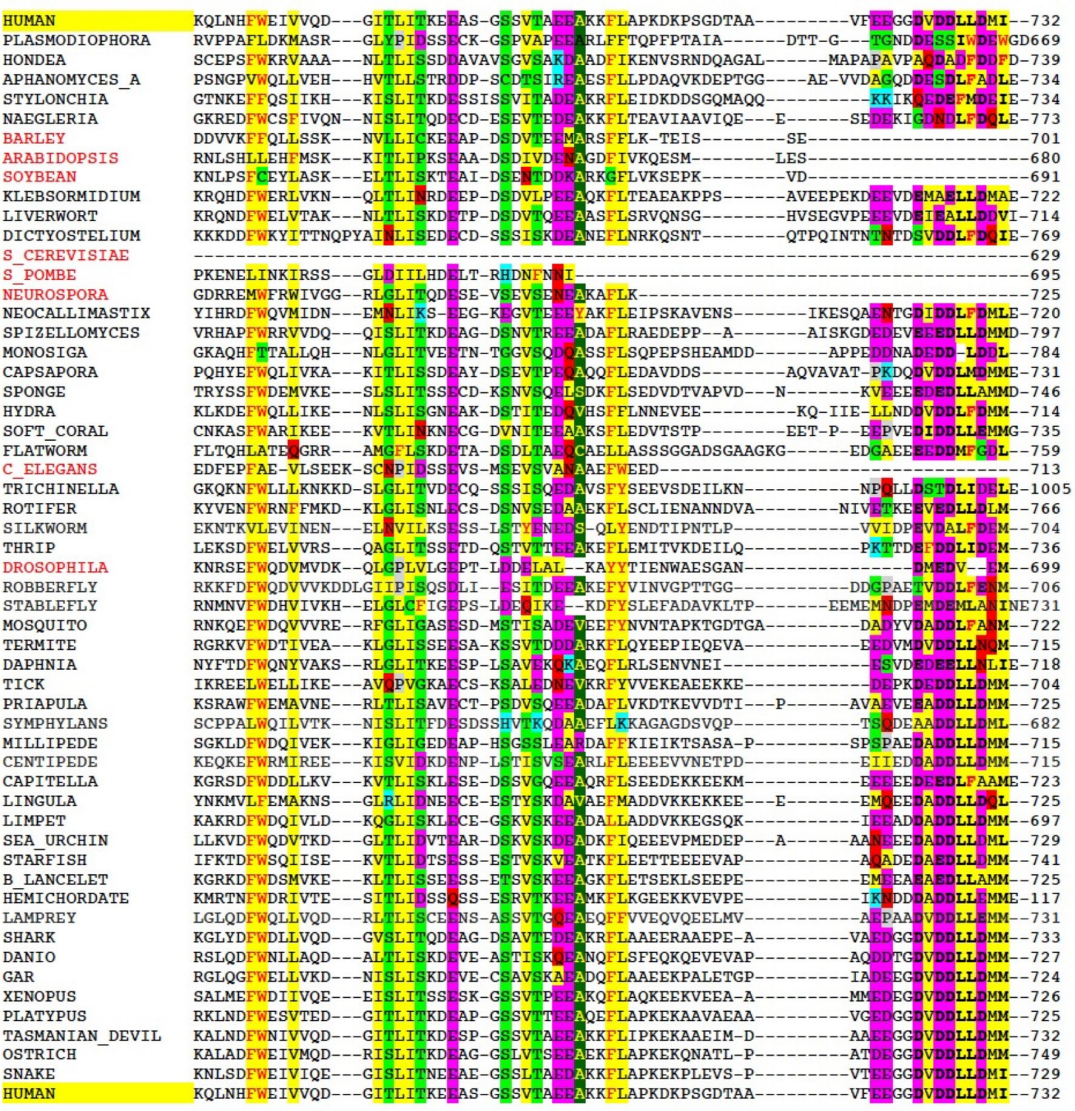
Conservation of the C-terminal region of Ku80 in vertebrates and multiple invertebrates and plants that also contain DNA-PKcs, but not in Dikarya yeast, *C. elegans* and flowering plants that do not. Multiple protein sequence alignment of Ku80 was carried out using Clustal Omega and conserved amino acids were colored manually using the following criteria: acidic amino acids (D, E) are highlighted in magenta; basic amino acids (R, K, H) are highlighted in blue; small polar amino acids (T, S, G, C) are in green; aliphatic amino acids (A, L, M, I, V) are in yellow; large polar amino acids (Q, N) are in red; aromatic amino acids (F, W, Y) are in red with yellow highlight; and proline (P) is in grey. Conservation of small amino acids (A, S, T, C, V, G) is illustrated by yellow letters with dark green highlight. In bold are the amino acids present in the structure of the long-range complex (DVDDLLDM) that interact with DNA-PKcs. Organisms and accession numbers for the sequences shown are as follows: PLASMODIOPHORE (Club root pathogen), *Plasmodiophora brassicae*, CEO94558.1; HONDAEA (Protist) *Hondaea fermentalgiana*, BG27299.1; APHANOMYCESa (Crayfish plague mold) *Aphanomyces astaci*, XP_009833210.1; STYLONYCHIA (Ciliate) *Stylonychia lemnae*, CDW74323.1; NAEGLERIA (Amoeba/flagellate), *Naegleria gruberi strain*, XP_002672413.1; BARLEY, *Hordeum vulgare*, AEO86624.1; ARABIDOPSIS, *Arabidopsis thaliana*, NP_564520.1; SOYBEAN, *Glycine max*, XP_003524779.1; GREEN_ALGAE, *Klebsormidium nitens*, GAQ85406.1; LIVERWORT, *Marchantia polymorpha*, PTQ30121.1; DICTYOSTELIUM, *Dictyostelium purpureum*, XP_637846.1; S_CEREVISIAE, *Saccharomyces cerevisiae*, NP_013824.1; S_POMBE, S*chizosaccharomyces pombe*, Q9HGM8.1; NEUROSPORA, (Bread mold), *Neurospora crassa*, AFM68948.1; NEOCALLIMASTIX (Anaerobic gut fungi), *Neocallimastix californiae*, ORY73184.1; SPIZELLOMYCES (Chytridomycota fungus), *Spizellomyces punctatus*, KND04299.1; MONOSIGA, (Choanoflagellate), *Monosiga brevicollis*, XP_001750285.1; CAPSASPORA (Amoeboid protist), *Capsaspora kowczarzaki*, KJE95979.1; SPONGE, *Amphimedon queenslandica*, XP_019851059.1; HYDRA, *Hydra vulgaris*, XP_012555181.1; SOFT_CORAL, *Dendronephthya gigantea*, XP_028404243.1; FLATWORM, *Macrostomum lignano*, PAA63650.1; C_ELEGANS, *Caenorhabditis elegans*, CAA83623; TRICHINELLA, Parasitic roundworm, *Trichinella pseudospiralis*, KRZ25527.1; ROTIFER, *Brachionus plicatilis*, RNA39402.1; SILKWORM, *Bombyx mori*, XP_037875756.1; THRIP, *Frankliniella occidentalis*, XP_026281764.1; DROSOPHILA, *Drosophila melanogaster*, NP_609767.2; ROBBERFLY, *Eutolmus rufibarbis*, SRR5185497; STABLEFLY, S*tomoxys calcitrans*, XP_013115123.1; MOSQUITO, *Aedes aegypti*, XP_001657128.2; TERMITE, *Cryptotermes secundus*, XP_023718673.1; DAPHNIA, *Daphnia magna*, XP_032789667.1; TICK, *Ixodes scapularis*, XP_002405506.2; PRIAPULA, Penis worm, *Priapulus caudatus*, XP_014668438.1; MILLIPEDE, *Craspedosoma sp.*, GERS01021842.1; CENTIPEDE, *Himantarium gabrielis*, GCIL01016305.1; CAPITELLA, Annelid worm, *Capitella teleta*, ELU08335.1; LINGULA, Brachiopod, *Lingula anatine*, XP_013403161.1; LIMPET, *Lottia gigantea*, XP_009064003.1; SEA URCHIN, *Strongylocentrotus purpuratus*, XP_788472.3; STARFISH, *Acanthaster planci*, XP_022103565.1; LANCELET, *Branchiostoma belcheri*, XP_019618779.1; HEMICHORDATE, Acorn worm, *Saccoglossus kowalevskii*, XP_006822840.1; LAMPREY, *Petromyzon marinus*, xp_032818808; SHARK, *Prionace glauca*, GFYY0116112.1; DANIO, *Danio rerio*, NP_001017360.2; GAR, *Lepisosteus oculatus*, XP_015214714.1; XENOPUS, *Xenopus laevis*, BAA76954.1; PLATYPUS, *Ornithorhynchus anatinus*, XP_028928834.1; TASMANIAN_DEVIL, *Sarcophilus harrisii*, XP_031813944.1; OSTRICH, *Struthio camelus australis;* XP_009666109.1*; SNAKE*,* Pseudonaja textilis*, XP_026563791.1. The names of organisms that do not contain DNA-PKcs are shown in red font. All others contain DNA-PKcs. See (Lees-Miller et al., 2020) for details. The human sequence is shown at the top and bottom in yellow highlight for comparison

Biochemical studies revealed that DNA-PKcs interacts with Ku via a conserved motif within the sequence EEGGDVDDLLDMI (residues 720–732) at the extreme C-terminus of Ku80 (Falck et al., 2005; Gell & Jackson, 1999). This DNA-PKcs interaction motif is attached to the body of Ku80 via a flexible linker (Hammel et al., 2010; Harris et al., 2004) and may be important not only for recruiting DNA-PKcs to the DSB but also for tethering autophosphorylated DNA-PKcs to the synaptic complex after its dissociation from the DSB ends (Fig. 2f). Although the linker was not visible in our structure, Ku80 residues 725–731 formed a discrete alpha-helix that interacted with the NUC194 domain of DNA-PKcs (residues 1815–2202) (Fig. 5). Low-resolution in this area of DNA-PKcs prevented assignment of interacting residues with certainty, but, from our structure it is likely that the DNA-PKcs helix between residues 1912–1923 as well as amino acids 1955–1970 are involved in the interaction with Ku80 CTR (Chen et al., 2021a). This assignment differs slightly from the Ku80-CTR-interacting regions identified in the recent structures of DNA-PKcs-Ku-DNA complexes (Chaplin et al., 2021; Chen et al., 2021b) perhaps indicating subtle differences in conformation of DNA-PKcs and Ku alone versus in the XLF-XRCC4-DNA Ligase IV complex (see also Hammel & Tainer, 2021; Hammel et al., 2020). Nevertheless, each of the structures reveals the importance of the NUC194 domain in the DNA-PKcs–Ku interaction. The NUC194 domain is conserved through evolution and is a signature of DNA-PKcs (Lees-Miller et al., 2020). Similarly, the Ku80 C-terminal motif DVDDLLDM is conserved in vertebrates, invertebrates, ciliates, molds and non-flowering plants that contain DNA-PKcs, but is absent from Dikarya yeast, flowering plants and organisms that lack DNA-PKcs (Figs. 5 and 6), suggesting that these regions co-evolved and that the function of DNA-PKcs in NHEJ is conserved in most eukaryotes. Interestingly, although *Drosophila* does not have DNA-PKcs/PRKDC, it retains a partial DNA-PKcs-interacting motif (DMEDVEM). This may relate to relatively recent loss, in evolutionary terms, of DNA-PKcs/PRKDC from *Drosophila* as it is present along with a conserved Ku80 COOH-terminus in other flies from the infra order Muscomorpha including the stable fly and robber fly. This is compared to loss in yeast at the sub-Kingdom level and flowering plants and Chromadorean nematodes at the class level.

Our cryo-EM structures revealed that in the absence of DNA-PKcs, Ku, dsDNA, XLF and X4-L4 formed a complex in which the two Ku heterodimers face each other, positioned by the XRCC4-DNA Ligase IV-XLF-XRCC4-DNA Ligase IV scaffold, such that the DSB ends are horizontal and aligned (Chen et al., 2021a). We termed this a short-range synaptic complex, in reference to earlier FRET-based studies in *Xenopus* egg extracts (Graham et al., 2016, 2018). Significantly, in this complex, the 16 bp of DNA previously held within the cradle domain of DNA-PKcs is now free, providing an ideal substrate for efficient nick sealing by Ligase IV (S. Chen et al., 2021a). We propose that DNA-PKcs acts as a “molecular ruler”, initially drawing ends of the DSB into its central cavity until further passage is blocked by interaction with the DEB. In this conformation, the ends of the DSB are slightly opened, or melted, which may be important for subsequent end-processing and ligation. Based on these structures and the in vitro and cell-based data described above, we propose that DNA-PKcs autophosphorylation triggers transition from the long-range to the short-range synaptic complex (Chen et al., 2021a). Moreover, our structures further show that Ligase IV ligates one nick within the DSB at a time in a sequential manner, suggesting a unique catalytic mechanism for rejoining (Chen et al., 2021a) (Fig. 2h–j). This finding is also able to explain how the unusual single turn-over DNA ligase, DNA Ligase IV, is able to completely ligate two nicks in DSBs via the NHEJ pathway (Chen et al., 2009).

## Future considerations

The structures of these NHEJ synaptic complexes provide unprecedented insight into the mechanism of NHEJ and also raise new questions. The location of the adjacent DNA-PKcs molecules such that the kinase domain of one DNA-PKcs molecule is adjacent to the forehead and concave ring of the opposite and partially inverted DNA-PKcs, places the one DNA-PKcs active site in position to phosphorylate the disordered ABCDE loop of the opposite molecule in trans (Fig. 3). This observation supports our hypothesis that autophosphorylation of the ABCDE cluster plays an important role in disassembly of the complex. For example, phosphorylation of the ABCDE cluster would introduce negative charges that could disrupt the interaction of the positively charged DEB helix with the ends of dsDNA (Fig. 4). However, DNA-PKcs autophosphorylation is more complex than just ABCDE phosphorylation, involving phosphorylation of the PQR cluster, and perhaps other sites, and we cannot exclude the possibility that phosphorylation at some sites may occur in cis as well as in trans. Ablation of the ABCDE and PQR phosphorylation sites has opposing effects on end-processing, with ABCDE promoting end-processing and PQR limiting it (Cui et al., 2005; Ding et al., 2003).

A recent study suggests that autophosphorylation occurs in a two-step process where different types of DNA promote autophosphorylation at different sites and affects the ability to phosphorylate exogenous substrates. For example, dsDNA with closed DNA hairpin ends (mimicking DNA produced by RAG endonucleases at coding ends in V(D)J recombination) supports robust phosphorylation of T2609 in the ABCDE cluster with minimal phosphorylation of either S2056 in the PQR cluster or exogenous substrates such as p53, Hsp90 and XRCC4. In contrast, dsDNA with open ends supports phosphorylation of T2609, S2056 and exogenous substrates (Meek, 2020). These findings have important implications not only for the mechanism of activation of DNA-PKcs and thus regulation of NHEJ, but also how DSB ends are processed during V(D)J recombination. The data suggests that in the first step of phosphorylation, RAG-induced DNA hairpins on coding ends support ABCDE phosphorylation, resulting release of DSB ends by DNA-PKcs, permitting activation of the hairpin opening activity of Artemis. Once the hairpins have been opened, the free DNA ends support phosphorylation of S2056 and full activation of DNA-PKcs (Meek, 2020).

It has also been reported that the related PIKKs ATM and ATR can phosphorylate DNA-PKcs on the ABCDE sites (Chen et al., 2007; Jiang et al., 2015; Yajima et al., 2006). How this would occur in the context of the synaptic complex described here is unclear, perhaps indicating that ATM-dependent phosphorylation of DNA-PKcs occurs when DNA-PKcs and Ku assemble on DSB ends but are unable to form a synaptic complex. In addition, from our model we suggest that once DNA-PKcs has undergone autophosphorylation it dissociates from the DSB ends. For it to re-form a synaptic complex and/or to be phosphorylated by other PIKKs, it seems likely that the ABCDE/PQR sites would need to be dephosphorylated. Although we have shown that protein phosphatase 6 (PP6) interacts with DNA-PKcs, depletion of PP6 did not result in increased ABCDE phosphorylation suggesting that PP6 may not be responsible for dephosphorylation of DNA-PKcs on the ABCDE sites (Douglas et al., 2010); therefore, DNA-PKcs dephosphorylation could involve other protein phosphatases. Alternatively, autophosphorylation could act as a signal for degradation and turnover of DNA-PKcs at the DSB.

One site that is clearly phosphorylated by ATM in response to DNA damage is serine 3205 in the FAT domain (Neal et al., 2011; Schlam-Babayov et al., 2021). Although the precise region surrounding S3205 was missing from our structure, the immediately surrounding amino acids are solvent facing, consistent with 3205 being located on an exposed flexible loop suitable to be phosphorylated directly by ATM (Fig. 3). S3205 is also an in vitro autophosphorylation site (Douglas et al., 2002). Phosphorylation is unlikely to occur in cis, therefore perhaps is phosphorylated by a neighboring DNA-PKcs molecule in vitro. In addition, DNA-PKcs phosphorylates ATM in response to DNA damage, inhibiting its activity (Zhou et al., 2017). Again, it is difficult to imagine how this could occur within the closed configuration of the synaptic complex, suggesting that DNA-PKcs can act in other conformations or complexes, independent of the synaptic complex.

Critical to NHEJ is the processing of the DSB ends to remove non-ligatable end groups. Potential processing enzymes include the nuclease Artemis, which interacts with both DNA-PKcs (Ma et al., 2002) and DNA Ligase IV (Ochi et al., 2013) and may play a role in removing blocked DNA ends; PNKP that interacts with CK2-phosphorylated XRCC4 and removes 3ʹ-phosphates or phosphoglycolates and adds 5ʹ-phosphates to create ligatable end groups (Weinfeld et al., 2011), as well as the Pol X family of DNA polymerases that can fill in gaps at the DNA termini (Kaminski et al., 2020; Ramsden & Asagoshi, 2012). Other NHEJ factors whose roles are less defined include PAXX (Xing & Oksenych, 2019), APLF (Hammel et al., 2016), CYREN/MRI (Arnoult et al., 2017; Hung et al., 2018) and long-non-coding RNA such as LINP1 (Thapar et al., 2021) (Fig. 2g), though PAXX and LINP1 have both been shown to stabilize the initial synaptic complex between DNA-PKcs and Ku on DNA (Thapar et al., 2021; Wang et al., 2018).

To maintain genome integrity, it is critical that unrepaired DSB ends are not released prior to ligation, which could result in inappropriate joining and chromosomal aberrations. As seen in our structures, the Ku-XLF-XRCC4-Ligase IV flexible scaffold is a critical component of both long-range and short-range synaptic complexes. In the long-range complex, the DSB ends are protected by DNA-PKcs. We propose that after autophosphorylation-induced dissociation of DNA-PKcs from DSB ends, intramolecular “swivelling” of the Ku/XRCC4 scaffold results in the DSB ends being aligned and positioned for joining by the catalytic domain of Ligase IV (Chen et al., 2021a). Critically, the catalytic domain of Ligase IV is attached to the XRCC4 scaffold via its tandem BRCT domains and a flexible linker (Ochi et al., 2010, 2012, 2014). Similarly, PNKP is tethered to XRCC4 via its N-terminal Fork-head associated (FHA) domain and a flexible linker (Aceytuno et al., 2017; Bernstein et al., 2005). In this way, the catalytic domains of Ligase IV and PNKP can dynamically access the DSB ends while remaining integral components of the NHEJ machine through tethering to XRCC4. A similar mechanism may be at play for DNA-PKcs, which is flexibly attached via the conserved Ku80-CTR (Fig. 2c–g), and for other processing enzymes such as Aprataxin and PNKP-like protein, APLF which is flexibly attached to both Ku and chromatin (Hammel et al., 2010, 2016) and the pol X family polymerases which interact with Ku (Zhao et al., 2020b). Critically, the intimate, though dynamic and flexible, interaction of the various NHEJ proteins with the core NHEJ scaffold may allow for coordinated hand-offs and efficient repair, preventing the release of toxic repair intermediates that could lead to genome instability as proposed for the base excision repair pathway (Hitomi et al., 2007).

We envision that phosphorylation by other PIKKs plays a critical role in regulating dynamic responses within NHEJ synaptic complexes, as sites on PNKP, XRCC4, Artemis and APLF, as well as DNA-PKcs itself, can be phosphorylated in an ATM-dependent manner in response to DNA damage (Chen et al., 2005b; Hammel et al., 2016; Neal et al., 2011; Schlam-Babayov et al., 2021; Segal-Raz et al., 2011; Yu et al., 2003, 2008; Zolner et al., 2011). Moreover, the DNA-PK complex can regulate the nuclease activity of MRE11 (Deshpande et al., 2020), suggesting that the NHEJ machinery regulates MRN-initiated resection and pathway choice (Shibata et al., 2014). A better understanding of not only how the NHEJ complex is regulated but how it interacts with other components of the DNA damage response such as MRN will be critical to understanding cellular mechanisms that prevent genome instability and cancer. The combination of X-ray crystal (Sibanda et al., 2017) and cryo-EM (Chaplin et al., 2021; Chen et al., 2021a, b; Sharif et al., 2017; Yin et al., 2017) structures with solution dynamics by X-ray scattering (Hammel & Tainer, 2021; Hammel et al., 2020) will provide a means to guide ongoing studies to both test and understand the functional dynamics of DNA-PK complex in orchestrating NHEJ.

## Summary

In conclusion, the high-resolution structures of long-range and short-range synaptic complexes along with a new evolutionary analysis of DNA-PKcs sequence conservation provide unprecedented insight into the mechanism of NHEJ, the role of DNA-PKcs in synapsis and how DSBs are first detected, tethered then aligned for ligation. Conservation of the YRPD motif and the basic DEB-A/DEB helices suggests that the synapsis mechanism and DNA-PKcs-dependent autophosphorylation-induced regulation of NHEJ is conserved throughout evolution. DNA-PKcs is a potential cancer target, and small molecule DNA-PKcs inhibitors are currently in clinical trials (Cleary et al., 2020). The elucidation of the structure of these NHEJ synaptic complexes opens the door not only for a better understanding of NHEJ, but for design of advanced NHEJ inhibitors that could target allosteric conformations as well as its active site and thereby have improved specificity and therapeutic potential. Furthermore, the combination of advanced allosteric DNA-PK inhibitors enabled by this mechanistic understanding with focal radiation therapy may provide new opportunities for precision oncology.

## Data Availability

Sequences and structure codes were obtained from publicly accessible databases (NCBI and Protein Data Bank, respectively) as indicated in the text.
